# An Application of a Physiologically Based Pharmacokinetic Approach to Predict Ceftazidime Pharmacokinetics in a Pregnant Population

**DOI:** 10.3390/pharmaceutics16040474

**Published:** 2024-03-28

**Authors:** Khaled Abduljalil, Iain Gardner, Masoud Jamei

**Affiliations:** Certara Predictive Technologies, Level 2-Acero, 1 Concourse Way, Sheffield S1 2BJ, UK

**Keywords:** ceftazidime, pregnancy, feto-placenta, PBPK model, renal, GFR

## Abstract

Physiological changes during pregnancy can alter maternal and fetal drug exposure. The objective of this work was to predict maternal and umbilical ceftazidime pharmacokinetics during pregnancy. Ceftazidime transplacental permeability was predicted from its physicochemical properties and incorporated into the model. Predicted concentrations and parameters from the PBPK model were compared to the observed data. PBPK predicted ceftazidime concentrations in non-pregnant and pregnant subjects of different gestational weeks were within 2-fold of the observations, and the observed concentrations fell within the 5th–95th prediction interval from the PBPK simulations. The calculated transplacental clearance (0.00137 L/h/mL of placenta volume) predicted an average umbilical cord-to-maternal plasma ratio of 0.7 after the first dose, increasing to about 1.0 at a steady state, which also agrees well with clinical observations. The developed maternal PBPK model adequately predicted the observed exposure and kinetics of ceftazidime in the pregnant population. Using a verified population-based PBPK model provides valuable insights into the disposition of drug concentrations in special individuals that are otherwise difficult to study and, in addition, offers the possibility of supplementing sparse samples obtained in vulnerable populations with additional knowledge, informing the dosing adjustment and study design, and improving the efficacy and safety of drugs in target populations.

## 1. Introduction

Pharmacotherapy in obstetrics cannot always be avoided throughout pregnancy, and in many cases, it is needed to maintain maternal and/or fetal health. Understanding how the disposition of drugs changes during pregnancy is challenging as there are numerous continuous physiological changes taking place during pregnancy that can affect drug pharmacokinetics (PK) [1,2]. Selecting the appropriate dose to attain the desired pharmacological effect in pregnant subjects is challenging, and simply using the dose based on non-pregnant subjects can lead to sub-optimal therapy [3]. In addition, the presence of potential additional risks to the fetus, when using pharmacological interventions in the mother, adds another level of complexity when attempting to deliver safe and effective treatment to pregnant subjects [4,5]. Therefore, it is proposed that if knowledge of the physiological changes that occur during pregnancy can be used to provide some insight into potential PK alterations during pregnancy, this will be beneficial in guiding initial prescribing strategies to select an optimal dose to treat the mother whilst protecting the fetus.

Physiologically-based-pharmacokinetic (PBPK) modeling has been widely used to investigate the influence of physiological changes in different subjects, or in specific populations, on drug disposition [6,7,8]. The application of PBPK models to predict drug exposure in pregnant women is increasing due to their ability to integrate knowledge from different sources. This allows the inclusion of prior gestational age-related changes in physiological parameters together with information on the physicochemical properties, in vitro disposition information (binding, metabolism, permeability, solubility, etc.), and human PK of the drug to be considered in the PBPK model [1,9,10]. 

Among the key physiological changes occurring during pregnancy are changes in the renal function due to the continuous increase in the maternal glomerular filtration rate (GFR), cardiac output (CO), and renal blood flow (RBF) with the gestational time. These changes can, collectively, affect the pharmacokinetics of many drugs [11]. Meta-analyses and mathematical quantification of these changes during pregnancy have previously been described [2].

The aim of this work was to develop a PBPK model to describe the pharmacokinetics of ceftazidime in non-pregnant and pregnant subjects.

Ceftazidime is mainly administered as an i.v. or i.m. injection, and its PK largely depends on the subject’s renal function. Ceftazidime clearance is not affected by concomitant administration of probenecid, suggesting that the mechanism of the renal excretion of ceftazidime is mainly through glomerular filtration [12,13]. About 70% of a ceftazidime dose is excreted as unchanged in the urine within the first 2 to 4 h, reaching about 80–90% within 24 h [13]. Ceftazidime is commonly used to treat infection during pregnancy and was selected as a model drug for this exercise due to the availability of PK data. These features, in addition to its well-documented high transplacental transfer, provide an opportunity to assess whether a PBPK model using these data could be simulated and whether the changes in renal function during pregnancy could explain the observed differences in ceftazidime PK in clinical studies.

## 2. Materials and Methods

For all predictions of ceftazidime kinetics, the Simcyp Simulator V23 (Certara UK, Sheffield, UK) was used with the built-in models for non-pregnant and pregnant populations. The results from all simulations were compared to observed clinical data. No restrictions were applied to the observed clinical data whether ceftazidime concentration was measured by high-performance liquid chromatography (HPLC) or microbiological (MBA) assay. A total of 20 trials were used in each executed simulation using the reported sample size for each trial. If a clinical study used <10 subjects, a minimum of 10 subjects in 20 trials were used for these virtual trials (200 subjects) to get a better description of the derived PK parameter and its associated variability.

The physicochemical parameters used in the ceftazidime PBPK model are based on a previously published ceftazidime PBPK model [14] with the following two modifications: (a) the non-renal clearance was allocated to biliary clearance as the drug is known to be excreted in the bile [15,16], and (b) the fraction unbound in plasma (fu) of 0.9 was predicted within the Simcyp Simulator based on the compound pKa and LogP properties [17]. As per the original model [14], the distribution of ceftazidime into the tissues was described using a full-body PBPK model with tissue partition coefficients (Kps) being predicted according to Rodgers and Rowland [18]. The renal elimination of ceftazidime was described using renal passive filtration, whereby individuals’ GFR values were estimated within the Simulator according to the Cockcroft–Gault equation [19], taking into account the simulated individual’s body surface area [20].

(1) $$GFR\;\left(mL/min/1.73\;m^{2}\right)=\frac{\left(140-age\right)\;\cdot WT}{72\;\cdot Scr/88.42}\;\cdot \frac{1.73}{BSA}\cdot \left(0.82\;if\;female\right)$$

where age, *WT*, *Scr*, and *BSA* are the individual’s age in years, weight in kg, serum creatinine in µmol/L, and body surface area in m^2^. The impact of these modifications was verified against clinical data after a 1 g bolus dose [21,22]. The model was then extended to describe ceftazidime kinetics after an intramuscular (i.m.) injection using a lag time and a first-order rate constant for absorption. Values for these parameters were adjusted to recover observed data after a single dose of 0.5 g [22,23]. The full list of input parameters in the ceftazidime PBPK model is given in the Supplementary Materials Table S1. 

**Figure 1:**
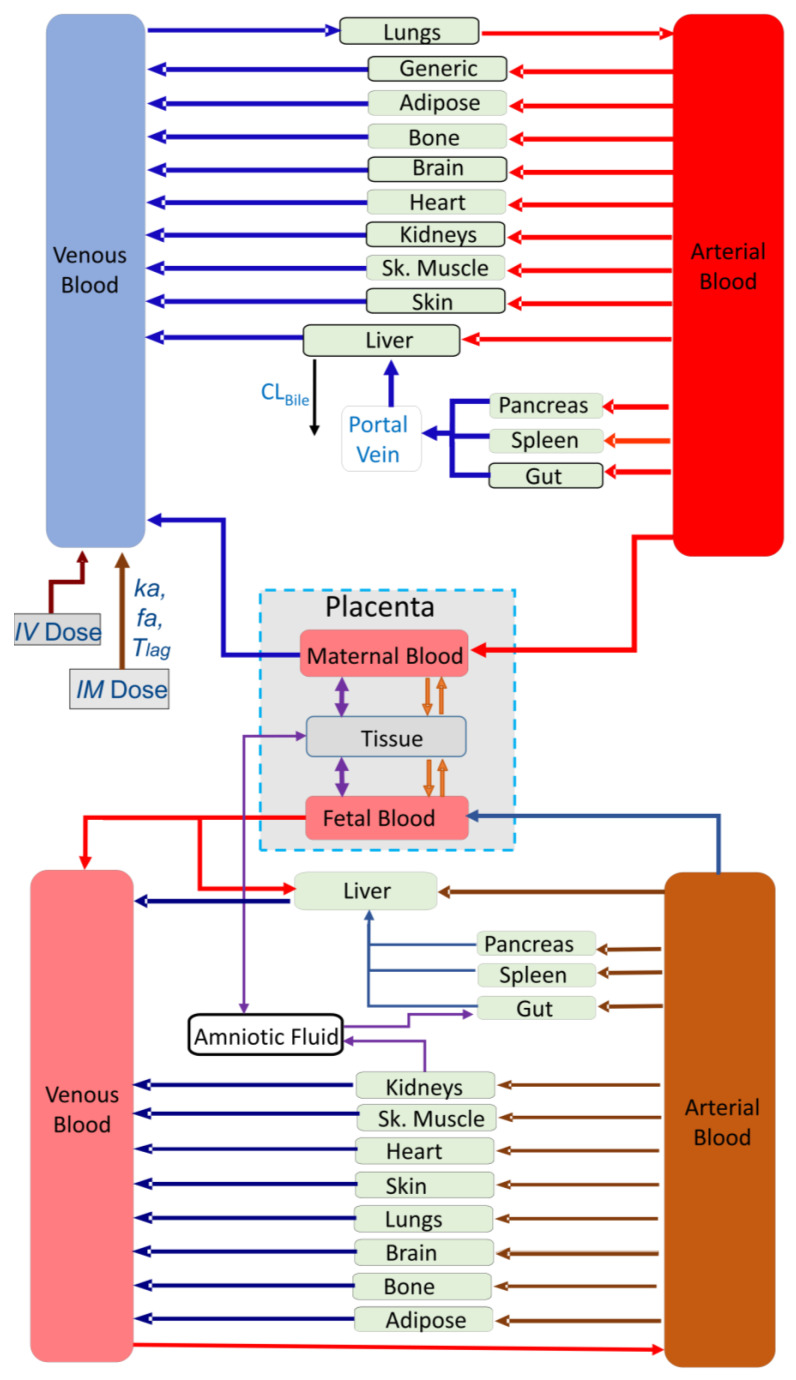
Ceftazidime pregnancy PBPK model structure.

Performance verification of this base model for predicting ceftazidime exposure in non-pregnant subjects was then carried out using the “Sim-Healthy Volunteers” population library within the Simulator after setting different dosing regimens mimicking published clinical studies after i.v. and i.m. administrations. Since the goal is to develop a model that describes exposure in pregnancy and most available data are from the Japanese pregnant population, the verification of the base model in non-pregnant subjects includes data from both Japanese and Caucasian populations. This base model for non-pregnant subjects was then extended to predict ceftazidime kinetics in pregnant subjects at different gestational weeks (GWs) by selecting the built-in pregnancy population within the Simulator. The changes in predicted values for GFR (*GFR_pred_*), CO (*CO_pred_*), and RBF (*RBF_pred_*) are described in the PBPK model using the following functions based on a previously published analysis [2]:

(2) $${GFR}_{pred}\left(\frac{mL}{min}\right)={GFR}_{\left(0\right)}*(1+0.028392\;GW-0.000502\;{GW}^{2})$$

(3) $${CO}_{pred}\left(\frac{L}{h}\right)={CO}_{\left(0\right)}*(1+0.019657\;GW-0.000292\;{GW}^{2})$$

(4) $${RBF}_{pred}\left(\%CO\right)={RBF}_{\left(0\right)}*(1+0.024453\;GW-0.00076\;{GW}^{2})$$

where ${GFR}_{\left(0\right)},{CO}_{\left(0\right)},\text{and}\;{RBF}_{\left(0\right)}$ are the baseline values in non-pregnant women [2,24,25]. Exposure in the fetus was simulated to occur via linking the maternal PBPK model to the multi-compartment feto-placental model (Figure 1). Growth of the placenta and fetal tissues and their blood flows as well as binding proteins were all gestational-dependent parameters within the model. More details on the feto-placental model assumptions and application have been described previously [25,26]. 

A ceftazidime transplacental clearance of 0.00137 L/h/g placenta tissue was calculated within the Simulator from ceftazidime physicochemical properties, namely, its hydrogen bond donor and topical polar surface area as described previously [25]. To predict the amniotic exposure of ceftazidime, the fetal renal clearance was predicted from the adult clearance and the maturation of fetal GFR as described earlier [25].

Clearances between the fetal tissue and amniotic fluid, as well as fetal swallowing, were accounted for in the fetal PBPK model as described previously [26]. The full list of the model input parameters is available in Table S1 of the Supplementary Material.

**Virtual trial settings:**

The virtual trial settings used for model building and for prediction of ceftazidime PK in non-pregnant subjects are given in Appendix A. The virtual trial settings used for the prediction of ceftazidime PK in pregnant subjects are given in Appendix B.

**Model application:**

The developed ceftazidime pregnancy PBPK model was used to assess different regimens that can maintain maternal and umbilical cord plasma levels above a minimum inhibitory concentration (MIC) of 8 mg/L, which is the clinical susceptibility breakpoint against many bacterial species, including *Enterobacteriaceae* and *Pseudomonas aeruginosa* [27]. The following scenarios were explored in virtual pregnant subjects with normal (Cases 1 to 4) and reduced (Cases 5 to 8) renal function for their gestational weeks at 20, 30, and 40 GWs:

Case 1: Three doses of 2 g bolus every 8 h to subjects with normal GFR.

Case 2: 2 g bolus and then 4 g as 23.5 h infusion to pregnant subjects with normal GFR.

Case 3: 2 g bolus and then 3 g to pregnant subjects with normal GFR. 

Case 4: 1 g bolus and then 3 g as 23.5 h infusion to pregnant subjects with normal GFR. 

Case 5: 1 g bolus and then 3 g as 23.5 h infusion to pregnant subjects with reduced (×0.75) GFR. 

Case 6: 0.5 g bolus and then 3 g as 23.5 h infusion to pregnant subjects with reduced (×0.75) GFR.

Case 7: 0.5 g bolus and then 3 g as 23.5 h infusion to pregnant subjects with reduced (×0.50) GFR.

Case 8: 0.5 g bolus and then 2 g as 23.5 h infusion to pregnant subjects with reduced (×0.50) GFR.

The bolus dose was given over 5 min, while the infusion started 30 min after the bolus dose.

**Assessment criteria:**

Depending on data availability, the predicted PK profiles and/or PK parameters were compared with different sets of clinical observations available in the literature. The PBPK model predictions were considered successful and acceptable if the observed PK profile fell within the 95th and 5th percentile of predicted data and the predicted PK parameters fell within 0.5- to 2-fold of the observed data.

## 3. Results

**Figure 2:**
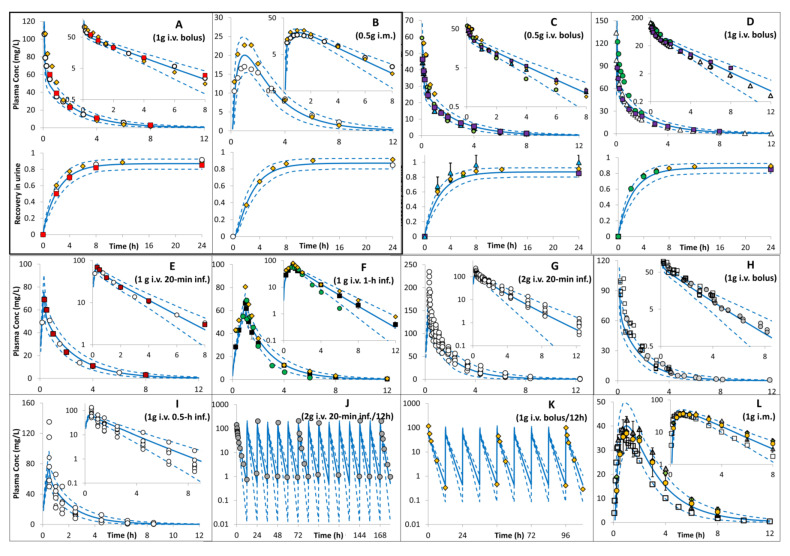
Plasma concentration profiles after intravenous and intramuscular administration of ceftazidime in non-pregnant subjects. Solid lines = predicted means, Dashed lines = 5th and 95th centiles. Data used for model development are shown in plot (**A**) (squares [13]; circles [21], and diamonds [22]) after i.v. bolus and in plot (**B**) i.m. (circles [23]; diamonds [22]) dose. Predictions against observation are shown in plot (**C**) (triangles [21], squares [23], diamonds [22], and circles [28]), plot (**D**) (triangles [29], squares [23], diamonds [22], and circles [28]), plot (**E**) (circles [23] and squares [13]), plot (**F**) (diamond [22], circle [28], and square [30]), plot (**G**) [31], plot (**H**) (circles [32] and squares [33]), plot (**I**) [34], plot (**J**) [15], plot (**K**) [22], and plot (**L**) (circles [21], triangles [35], squares [29], and diamonds [23]). Observed data are mean values, except for Warns et al., [31], Seiga et al., [32], Kohara et al., [33], and Doko et al., [34] were individual data were available. Error bars represent standard deviations. See Appendix A for trial settings.

Ceftazidime simulations for the baseline model in non-pregnant subjects are shown in Figure 2. The PBPK model predictions agreed with the observed data in different studies after the i.v. and i.m. administrations. Observed plasma concentrations and the cumulative fraction excreted in urine were within the simulated 5th–95th prediction interval. A comparison of the predicted PK parameters in the non-pregnant population with those available from the clinical studies is shown in Table 1, which also shows the agreement between the observed and the predicted PK parameter values.

**Figure 3:**
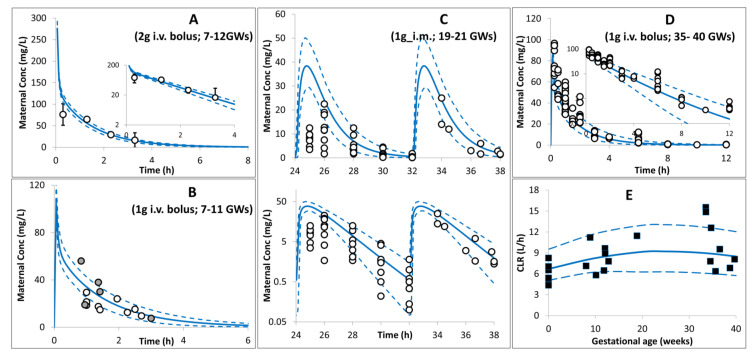
Maternal plasma concentration (**A**–**D**) and clearance (**E**) profiles after intravenous (**A**,**B**,**D**) and intramuscular (**C**) administration of ceftazidime in pregnant subjects at different gestational weeks (plot (**A**) 7–12 GWs [37]; plot (**B**): 7–11 GWs (open circles [38] and closed circles [39]), plot (**C**) [40], plot (**D**) [41], and plot (**E**) [42]). Solid lines = predicted means, Dashed lines = 5th and 95th centiles. Error bars in the first plot represent standard deviations. The rest of the observed data are individual values. See the Appendix B for trial settings.

**Figure 4:**
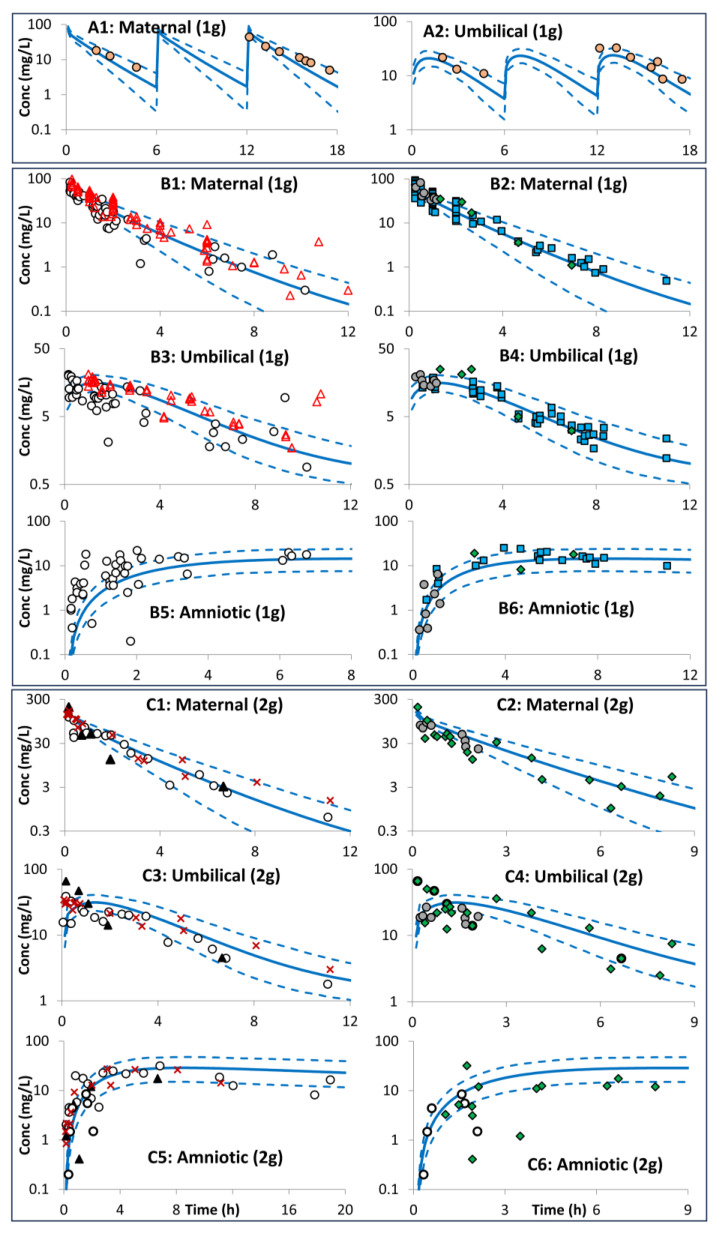
Maternal plasma, umbilical vein plasma, and amniotic concentration profiles after intravenous administration of ceftazidime in pregnant subjects at delivery. Plot (**A1**,**A2**) at 25–34 GWs [43], plot (**B1**–**C6**) at delivery > 37 GWs (open circles [44], open triangles [41], filled circles [45], diamonds [39], squares [46] closed triangles [38], and crosses [47]. (**B1**–**B6**) after single 1 g i.v. dose, and (**C1**–**C6**) after a single 2 g i.v. dose. Solid lines = predicted means, Dashed lines= 5th and 95th centiles. Observations are individual values from different studies. See Appendix B for trial settings.

Limited data were available during the first and second trimesters, with most of the available ceftazidime PK studies in pregnancy carried out toward the end of the third trimester and at the time of delivery. The PBPK model predictions for the different trimesters of pregnancy are shown in Figure 3 for maternal PK during pregnancy and in Figure 4 for the maternal, umbilical cord, and amniotic fluid concentration profiles at delivery. Available PK parameters from opportunistic sampling, where reported, were compared with model predictions in Table 2. The developed model predicted a mean cord-to-plasma AUC ratio at term of 0.73 ± 0.10 (range: 0.58–0.84) after the 1st dose, which increased to 0.93 ± 0.03 (range: 0.85–1.01) at a steady state.

**Figure 5:**
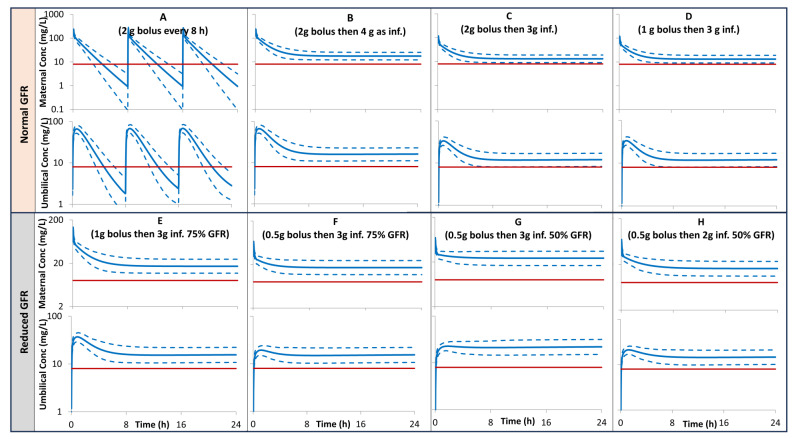
PBPK predictions for maternal and umbilical ceftazidime plasma concentration profiles in pregnant subjects at 20 GWs with 100% (**A**–**D**), 75% (**E**,**F**), 50% (**G**,**H**) of a normal GFR for their gestational week. Solid profiles = predicted means, Dashed profiles = 5th and 95th centiles. Horizontal lines represent MIC of 8 mg/L. inf. = infusion. Plots (**A**–**H**) correspond to Cases 1–8, respectively (See Materials and Methods section).

**Figure 6:**
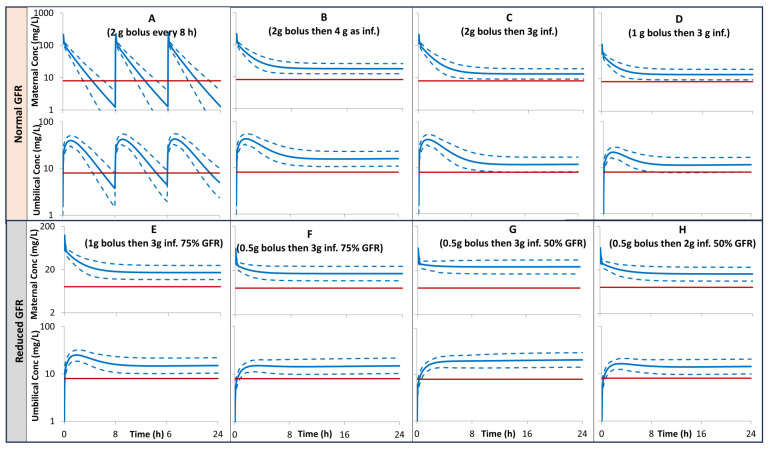
PBPK predictions for maternal and umbilical ceftazidime plasma concentration profiles in pregnant subjects at 30 GWs with 100% (**A**–**D**), 75% (**E**,**F**), 50% (**G**,**H**) of a normal GFR for their gestational week. Solid profiles = predicted means, Dashed profiles = 5th and 95th centiles. Horizontal lines represent MIC of 8 mg/L. inf. = infusion. Plots (**A**–**H**) correspond to Cases 1–8, respectively (See Materials and Methods section).

**Figure 7:**
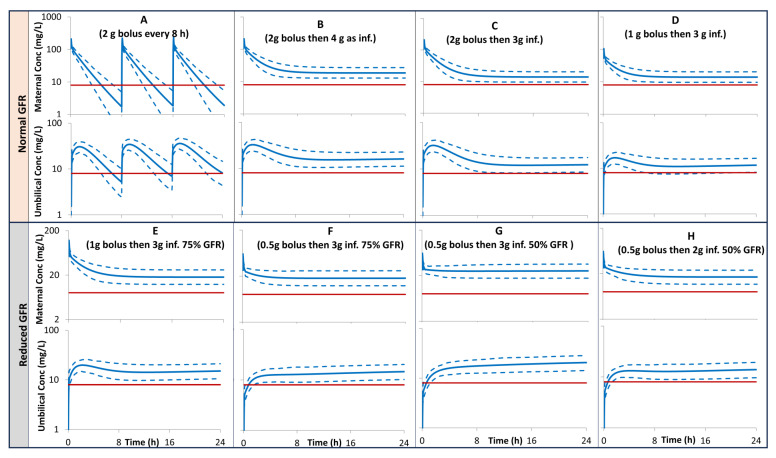
PBPK predictions for maternal and umbilical ceftazidime plasma concentration profiles in pregnant subjects at 40 GWs with 100% (**A**–**D**), 75% (**E**,**F**), 50% (**G**,**H**) of a normal GFR for their gestational week. Solid profiles = predicted means, Dashed profiles = 5th and 95th centiles. Horizontal lines represent MIC of 8 mg/L. inf. = infusion. Plots (**A**–**H**) correspond to Cases (1–8), respectively (See Materials and Methods section).

The model prediction for maternal and umbilical plasma concentration after different scenarios of multiple bolus doses vs. constant infusions performed for pregnant subjects with normal renal function, together with the MIC of 8 mg/L, a breaking point for most of the susceptible bacteria including *Pseudomonas aeruginosa*., are given in Figure 5, Figure 6 and Figure 7 for pregnant individuals at 20, 30, and 40 GWs, respectively. A loading dose of 1 g bolus followed by 3 g given as a constant infusion over 23.5 h is predicted to give both maternal and umbilical levels of >8 mg/L. In pregnant subjects with 50% lower GFR, a loading dose of 0.5 g bolus followed by 2 g given as a constant infusion over 23.5 h is predicted to give both maternal and umbilical levels of >8 mg/L.

## 4. Discussion

This work describes the use of a PBPK framework approach to describe the pharmacokinetics of ceftazidime during pregnancy. Drug concentrations were predicted in the mother and in the umbilical cord. Ceftazidime was chosen because of the data availability required for model building, because of the verification of its performance in non-pregnant and pregnant subjects, and because it is often used to treat and prevent infections during pregnancy and caesarian sections. Ceftazidime is mainly administered as an i.v. or i.m. injection, and its PK largely depends on the subject’s renal function. About 70% of the dose is excreted unchanged in urine within the first 2 to 4 h, and about 80–90% of the dose is excreted as unchanged ceftazidime in urine within 24 h [13]. The developed model assumes that the 24 h renal excretion accounts for about 88% (5th–95th percentiles: 80–92%) of the dose in non-pregnant healthy subjects (Figure S1 of the Supplementary Materials). The average mean value reported in different clinical studies indicated a wider range of 67–102% [21,36] in healthy adult subjects with normal renal function. The predicted plasma fu of 0.9 in non-pregnant subjects was within the published range from 0.77 to 1.0 [12,15]. 

The PBPK model predictions for ceftazidime PK in the non-pregnant population were in good agreement with the observed values after i.v. and i.m. administrations in different studies of variable dosing levels (Figure 2 and Table 1). Predicted PK parameters fall within 2-fold of the observed values. The predicted 5th and 95th percentiles for systemic exposure in plasma include the observed concentration versus time profiles.

The predicted enhanced renal function, that was observed during pregnancy, is expected to reduce ceftazidime systemic exposure to different magnitudes during pregnancy compared with non-pregnant subjects. The model predicted that the mean systemic (and renal) clearance would be 30% (39%) higher than the non-pregnancy value during the second trimester, and it declined slightly toward term but was still 22% (28%) higher than the non-pregnant level (boxplots of the fold increase from the non-pregnant lever are given in Figure S2 of the Supplementary Document). The considered increase in renal function within the pregnancy PBPK model adequately described the observed [42] increase in ceftazidime clearance during gestation (Figure 3). 

Observed data were available for ceftazidime exposure during delivery. The availability of these data facilitates the verification of the model predictions of the placental passage of ceftazidime. Figure 4 shows that the incorporation of the predicted ceftazidime transplacental clearance from its physicochemical properties resulted in an adequate prediction of the ceftazidime concentration in the umbilical cord when compared to the observed values. The developed model predicted a mean cord-to-plasma AUC ratio of 0.73 ± 0.10 (range: 0.58–0.84) after the 1st dose, which increased to 0.93 ± 0.03 (range: 0.85–1.01) at a steady state. This is in line with the observed data in clinical studies (Table 2). The developed feto-placental model predictions (Figure 4) showed that the umbilical cord plasma concentration peaked at an average of 1.5 h after the maternal maximum level was reached. Although the cord level decreases over time, it becomes higher than the maternal serum concentration after 4 h. In contrast, the amniotic level reaches its maximum after around 6 h. After 3 h of drug administration, there was no significant difference in the maternal blood and umbilical cord blood concentrations. 

The developed ceftazidime pregnancy PBPK model could be applied to assess different scenarios that can maintain the plasma exposure at or above 8 mg/L, which covers the most common non-resistant bacteria, such as *Enterobacteriaceae* and *Pseudomonas aeruginosa* [27]. The proposed exposure after long infusions was compared with the administration of 2 g every 8 h, a clinical dosing that has been evaluated in healthy male subjects [48]. These figures show that a lower daily dose of 4 g (1 g bolus followed, after 30 min, by a constant infusion of 3 g over 23.5 h) is sufficient to maintain the exposure at a value ≥ 8 mg/L. This dosing strategy has the advantage of reducing the maximum exposure and minimizing the degree of fluctuation over the treatment period. Whilst this was assumed for pregnant subjects at term, it can also provide protection pre-term for caesarian section procedures or for preventing cross-infection with susceptible bacteria. It can also give maternal protection in non-obstetric surgery. While the assumption here is that the patients have normal renal function, the total dose for pregnant women with renal impairment should be lower depending on their renal function. 

The slight underprediction of ceftazidime exposure in the simulated subjects compared with Dallmann’s study [43] is probably because the observations stemmed from pregnant subjects under mechanical ventilation. A previous study found that mechanical ventilation decreases ceftazidime renal clearance and increases its volume of distribution [49]. The over-prediction of plasma concentrations observed in Giamarellou’s study [40] is probably because the absorption in that clinical study was low for the first dose and also because the absorption model used in the current study does not account for changes to the blood flow at the injection site during pregnancy.

This work shows a successful prediction of maternal and umbilical exposure by accounting mainly for the change in renal function during pregnancy together with the integration of the chemical properties of ceftazidime; however, there are limitations to the study. Maternal data in the Caucasian studies are from pregnant subjects with comorbidity, and such elements are not part of the current model. Predictions for all pregnant subjects were made using Caucasian physiology since a pregnancy model specific to the Japanese population has not been developed yet. The gestational weight gain at the end of gestation was reported to be lower for low-risk Japanese pregnant subjects (10.9 kg; interquartile range (IQR), 8.7–13.2 kg [50]) compared with their Caucasian peers of 13.7 kg (IQR, 10.9–16.9 kg [51], and 14.5 kg (IQR, 11.5–17.7 kg [52]). Part of this difference is attributed to the lower feto-placental weight observed in the Japanese population. Both Japanese birthweight and placental weight are slightly lower (about 90%) than the birthweight in the Caucasian population [53,54]. These observations suggest that further work is required to assess whether a PBPK model for pregnant Japanese subjects needs to be built. The non-renal clearance of ceftazidime was described by a generic intrinsic biliary clearance and was not assigned to any specific transporter in the liver due to the lack of transporter kinetics data. Since this generic pathway constitutes <15% of the total clearance, the biliary intrinsic clearance was assumed to remain constant during pregnancy because it is challenging to define if it is induced or inhibited during pregnancy. No enterohepatic recirculation was included in the model since ceftazidime is not absorbed after oral administration. It is unlikely that this biliary pathway can influence the clearance of ceftazidime significantly since the major pathway via GFR is further enhanced during pregnancy. On the other hand, current clinical observations were collected opportunistically as these clinical studies were not designed to investigate the impact of pregnancy at different gestational weeks on ceftazidime PK, neither on the maternal nor on the fetal level. Due to the nature of opportunistic sampling of the plasma only, and not being previously powered, many reported PK parameters from these studies (presented in Table 2) were limited in nature. In addition, none of the clinical studies during pregnancy reported the cumulative amount of ceftazidime excreted in urine. The absence of a control arm, i.e., ceftazidime PK in healthy pregnant subjects, makes it challenging to delineate if there is any impact of comorbidity on ceftazidime PK during pregnancy. The developed model treats observations equally regardless of the analytical methods (HPLC or MBA) used in the original study as there is no overall trend for one method being higher or lower than the other. Harding et al. [21] indicated that HPLC and MBA assays gave essentially the same results with correlation coefficients of 0.93 to 0.99. Other studies also reported that the two analytical methods showed an excellent correlation for drug concentrations in urine with a slope of 1 and a correlation coefficient of 0.99 [15,23]. This was also the case for serum concentrations above 20 mg/L. Serum concentrations below 20 mg/L were found to be up to 30% smaller by HPLC than by bioassay in one study [15]; however, another study found that there was less than 1 mg/L difference between the assays for serum concentrations over the range of 9–22 mg/L, but at higher concentrations, the HPLC assay gave higher values than MBA [23]. These results indicate the lack of a consistent difference between MBA and HPLC.

Different scenarios of loading and maintenance doses were assessed and compared with an MIC of 8 mg/L to evaluate the lowest possible dosing regimen that can be administered in clinical awards, whilst retaining effectiveness (Figure 5, Figure 6 and Figure 7). The dosage of 1 g bolus followed by 3 g administered as a constant infusion over 23.5 h is predicted to give both maternal and umbilical exposure at a level > 8 mg/L, a breaking point for most of the susceptible bacteria including *Pseudomonas aeruginosa* (plot D in Figure 5, Figure 6 and Figure 7). This predicted exposure assumed normal renal function for the pregnancy at term. In pregnant subjects with low renal function, the daily dose is expected to be lower depending on the level of renal insufficiency. For example, similar bacterial protection can be achieved by 0.5 g bolus followed by 3 g infusion in pregnant individuals with 75% GFR (plot F) or 0.5 bolus followed by 2 g in individuals with 50% GFR (plot H).

As with any model, a PBPK model can only be as good as the underlying physiological data used to construct the model. Any measurement errors in the underlying physiology data would be expected to impact the simulated results from the PBPK model. However, in this case, the model was able to reasonably capture all the observed clinical data despite any limitations in the observed data or underlying physiology. As the knowledge and confidence in PBPK model predictions increase, these models could be used at the bedside in parallel with current clinical practice. This would allow a better understanding of the predictive performance of the models and may reveal any limitations in the underlying physiological parameters. Testing the model in different scenarios and refining the model if needed will lead to more robust models in the future. Whilst the use of sophisticated desktop software at the bedside can be problematic, simplified web-based applications can be developed that use a limited number of input parameters specific to an individual subject. These parameters can then be fed into computational engines running in the Cloud, enabling individualized dosage predictions for specific patients to be attained in real-time.

## 5. Conclusions

A PBPK approach was adopted to evaluate the pharmacokinetics of ceftazidime from the general population and at different gestational weeks throughout pregnancy. Utilizing a PBPK approach in special populations reinforces the utility of PBPK to assess pharmacokinetics in clinical settings where clinical data are limited and can be used to improve and inform the dose selection and study design in these vulnerable populations.

## Figures and Tables

**Table 1:** Predicted vs. observed ceftazidime PK parameters in non-pregnant healthy adult subjects.

Clinical Study		AUC (h mg/L)			C_max_ (mg/L)			Half-Life (h)		
Dose	Population	Obs	Pred	Ratio	Obs	Pred	Ratio	Obs	Pred	Ratio
0.5 g i.v. bolus	4 M: 20–49 yr [21]	71	74 ± 13	1.0	65.8	68.4 ± 8.5	1.0	1.5	1.7 ± 0.3	1.1
	8 M: 20–49 yr [23]	72 ± 5		1.0	57.6 ± 11		1.2	1.9 ± 0.6		0.89
	3 M: 31 yr [22]	83.6		0.9	73.4		0.9	1.74 ± 0.4		0.98
	*n* = 14 [28]	NA		NA	NA		NA	1.6		1.1
1 g i.v. bolus	4 M: 20–49 yr [21]	144	148 ± 25	1.0	121	149 ± 19	1.2	1.80	1.7 ± 0.3	0.94
	8 M: 20–49 yr [23]	136 ± 27		1.1	119 ± 27		1.2	1.8 ± 0.3		0.94
	3 M: 31 yr [22]	163		0.9	123		1.2	1.7 ± 0.3		1.0
	8 M: 22–24 yr [29]	133 ± 27		1.1	139 ± 33		1.1	1.64 ± 0.1		1.0
	*n* = 29 [28]	NA		NA	NA		NA	1.6		1.1
1 g 20 min i.v. inf	8 M: 20–49 yr [23]	143 ± 13	148 ± 25	1.0	72.1 ± 3.6	75.6 ± 9.4	1.0	1.9 ± 0.3	1.7 ± 0.3	0.89
1 g i.v. inf for 1 h	6 M: 20–23 yr [30]	157	163 ± 23	1.0	66.3 ± 3.1	66.8 ± 5.1	1.0	1.62 ± 0.12	1.5 ± 0.2	0.94
	3 M: 27–29 yr [22]	NA		NA	80.6		0.8	1.95 ± 0.26		0.77
	*n* = 7 [28]	NA		NA	69		1.0	1.64		0.91
2 g/8 h i.v. inf 20 min *	4 M/4 F: 20–30 yr [15]	297 ± 45	267 ± 55	0.90	201 ± 21	168 ± 23	0.84	1.84 ± 0.3	1.4 ± 0.3	0.77
1 g/12 h i.v. bolus *	3 M: 23–27 yr [22]	180	162 ± 23	0.90	108 ± 6.0	110 ± 12	1.0	1.6 ± 0.1	1.5 ± 0.2	0.94
1 g i.v. bolus	8 M/8 F: 20–45 yr [36]	156 ± 11	134 ± 27	0.87	146 ± 9	158 ± 22	1.1	1.45 ± 0.4	1.42 ± 0.4	1.0
0.5 g i.m.	8 M: 20–49 yr [23]	79 ± 8	74 ± 13	0.94	17.4 ± 2.5	20.4 ± 3.1	1.2	2.2 ± 0.3	1.7 ± 0.3	0.77
	3 M: 23–35 yr [22]	83.2		0.89	23		0.89	1.61 ± 0.2		1.1
1 g i.m.	8 M: 20–49 yr [21]	154.3	147 ± 25	0.95	37.2	40.7 ± 6.2	1.1	1.70	1.7 ± 0.3	1.0
	8 M: 20–49 yr [23]	175 ± 23		0.84	38.8 ± 4.5		1.0	2 ± 0.3		0.85
	8 M: 22–24 yr [29]	120 ± 17		1.2	43.2 ± 11		0.94	1.65 ± 0.1		1.0
1 g i.m.	8 M/8 F: 20–45 yr [36]	145 ± 9	133 ± 27	0.92	33.1 ± 1.6	42.4 ± 7.3	1.3	1.87 ± 0.4	1.4 ± 0.4	0.76

Abbreviations: i.v.: intravenous, inf: infusion, i.m.: intramuscular, AUC: area under the curve either at steady state or extrapolated to infinity after the single dose. CL: clearance, C_max_: maximum plasma concentration; Obs: observed; Pred: predicted. * Average of multiple doses are reported as there were no significant differences in PK parameters between the first and last doses.

**Table 2:** Predicted and observed ceftazidime PK parameters in pregnant subjects with normal renal function after intravenous administration.

Available Study Design Details		GWs	AUC (mg/L × h)			Half-Life (h)			Cord/Maternal Ratio		
			Obs	Pred	Ratio	Obs	Pred	Ratio	Obs	Pred	Ratio
Maternal	2 g bolus; *n* = 18: 18–26 yr [37]	7–12	231.8	207 ± 34	0.89	1.4	1.1 ± 0.2	0.79	NA	NA	NA
	1 g bolus; *n* = 12 F * [38]	7–11	NA	110 ± 19	NA	NA	1.0 ± 0.2	NA	NA	NA	NA
	1 g bolus; *n* = 7 F * [39]	7–11	NA	110 ± 19	NA	NA	1.0 ± 0.2	NA	NA	NA	NA
	1 g bolus/6 h; *n* = 10 [43]	26–34	110	103 ± 23	0.90	1.78	1.2 ± 0.2	0.68	NA	NA	NA
	1 g bolus; *n* = 28: 20–41 yr [41]	35–40	143	121 ± 46	0.85	1.37	2.0 ± 0.5	1.5	NA	NA	NA
	1 g bolus; *n* = 27: 20–41 yr [41]	At term	151	109 ± 24	0.7	1.4	2.0 ± 0.5	1.4	NA	NA	NA
	1 g bolus; *n* = 156 [38] *	At term	96.6	109 ± 24	1.1	1.44	2.0 ± 0.5	1.4	NA	NA	NA
	1 g 0.5 h inf.; *n* = 15 [38] *	At term	77.3	109 ± 24	1.4	1.24	2.0 ± 0.5	1.6	NA	NA	NA
	2 g bolus; *n* = 62 [38] *	At term	191	216 ± 48	1.1	1.4	2.0 ± 0.5	1.4	NA	NA	NA
Umbilical	1 g bolus/6 h; *n* = 10 [43]	26–34	121	87 ± 20	0.72	1.89	1.6 ± 0.3	0.85	1.0	0.85 ± 0.03	0.85
	1 g bolus; *n* = 27: 20–41 yr [41]	At term	85.7	86 ± 19	1.0	1.37	5.5 ± 2.1	4.0	0.67	0.73 ± 0.1	1.1
	1 g bolus; *n* = 161 [38] *	At term	95.4	86 ± 19	0.90	3.9	5.5 ± 2.1	1.4	0.99	0.73 ± 0.10	0.74
	1 g 0.5 h inf.; *n* = 15 [38] *	At term	74.5	86 ± 19	1.15	3.3	5.5 ± 0.21	1.67	0.96	0.73 ± 0.1	0.76
	2 g bolus; *n* = 66 [38] *	At term	143	173 ± 38	1.2	3.13	5.5 ± 2.1	1.75	0.75	0.73 ± 0.10	0.97

* age range was not mentioned, Obs: observed, Pred: predicted. NA: not applicable.

## Data Availability

The data presented in this study are available upon written request from the corresponding authors. Workspaces used to generate these results are available on request and can be found in the Certara Members Area.

## Supplementary Materials

The following supporting information can be downloaded at: https://www.mdpi.com/article/10.3390/pharmaceutics16040474/s1, Table S1: Ceftazidime PBPK model input parameters; Table S2: Collected clinical data on fraction of the dose excreted in the urine; Figure S1: Fraction of ceftazidime excreted as unchanged ceftazidime in urine; Figure S2: Fold change in systemic and renal clearance of ceftazidime at 10, 20, 30, and 40 GWs. Reference [55] is cited in the supplementary materials.

## Appendix A

**Virtual trial settings for ceftazidime PK in non-pregnant subjects:**

The following virtual trial settings were used for model building (MB) in non-pregnant (NP) subjects after either an i.v. or i.m. injection:

Trial design MB1: an i.v. bolus of 1 g ceftazidime [21,22]; 20 trials of 10 subjects (0% female) aged 20–49 years.

Trial design MB2: an i.m. injection of 0.5 g ceftazidime [22,23]; 20 trials of 10 subjects (0% female) aged 20–49 years.

Trial design NP1: an i.v. bolus of 0.5 g ceftazidime [21,22,23,28]; 20 trials of 10 subjects (0% female) aged 20–49 years.

Trial design NP2: an i.v. bolus of 1 g ceftazidime [22,23,28,29]; 20 trials of 10 subjects (0% female) aged 22–24 years.

Trial design NP3: an i.v. infusion of 1 g ceftazidime administered over 20 min [23]; 20 trials of 6 subjects (0% female) aged 20–49 years.

Trial design NP4: an i.v. 1 h infusion of 1 g ceftazidime [22,28,30]; 20 trials of 10 subjects (0% female) aged 23–38 years.

Trial design NP5: an i.v. infusion of 2 g ceftazidime administered over 20 min [31]; 20 trials of 8 subjects (50% female) aged 20–30 years.

Trial design NP6: an i.v. bolus of 1 g ceftazidime [32,33]; 20 trials of 10 subjects (100% female) aged 35–51 years.

Trial design NP7: an intravenous 0.5 h infusion of 1 g ceftazidime [34]; 20 trials of 10 subjects (100% female) aged 33–61 years.

Trial design NP8: multiple i.v. infusion of 2 g ceftazidime administered over 20 min twice daily for eight days [15]; 20 trials of 8 subjects (50% female) aged 20–30 years.

Trial design NP9: a multiple i.v. bolus of 1 g ceftazidime twice daily for a total of 9 doses [22]; 20 trials of 10 subjects (0% female) aged 23–27 years.

Trial design NP10: an i.v. bolus of 1 g ceftazidime [36]; 20 trials of 16 subjects (50% female) aged 23–26 years.

Trial design NP11: an i.m. injection of 1 g ceftazidime [21,23,29,35]; 20 trials of 10 subjects (0% female) aged 20–49 years.

Trial design NP12: an i.m. injection of 1 g ceftazidime [36]; 20 trials of 16 subjects (50% female) aged 23–26 years.

## Appendix B

**Virtual trial settings for ceftazidime PK in pregnant subjects:**

The following trial designs were set for model prediction during pregnancy to match the clinical studies after i.v. or i.m. administration of ceftazidime:

Trial design P1: an i.v. dose of 2 g ceftazidime infused over 3 min [37]; 20 trials of 18 Caucasian pregnant women aged 18–26 years at 7–12 GWs.

Trial design P2: an i.m. dose of 1 g ceftazidime every 8 h [40]; 20 trials of 10 pregnant women aged 18–35 years at 19–21 GWs.

Trial design P3: three i.v. bolus doses of 1 g ceftazidime every 6 h [43]; 20 trials of 10 pregnant women aged 18–45 years at 26–34 GWs.

Trial design P4: an i.v. bolus dose of 0.4 g ceftazidime followed by a constant infusion for 4 h [42]; 20 trials of 10 pregnant women aged 18–45 years at 0, 9–13 and 37 GWs.

Trial design P5: an i.v. bolus doses of 1 g ceftazidime [38,39]; 20 trials of 10 pregnant women aged 18–45 years at 7–11 GWs.

Trial design P6: an i.v. bolus dose of 1 g ceftazidime [41]; 20 trials of 10 pregnant women aged 20–40 years at 35–40 GWs.

Trial design P7: an i.v. bolus dose of 1 g ceftazidime [38,39,41,44,45,46]; 20 trials of 20 pregnant women aged 20–41 years at 37–40 GWs.

Trial design P8: an i.v. bolus dose of 2 g ceftazidime [38,39,44,45,47]; 20 trials of 20 pregnant women aged 20–40 years at 36–40 GWs.
