# Supplementary material for: An Application of a Physiologically Based Pharmacokinetic Approach to Predict Ceftazidime Pharmacokinetics in a Pregnant Population
Source: Pharmaceutics. 2024 Mar 28;16(4):474. doi: 10.3390/pharmaceutics16040474 (PMC11054561; doi:10.3390/pharmaceutics16040474)
Supplement: Supplementary file 1 [file pharmaceutics-16-00474-s001.zip › pharmaceutics-2916633-supplementary.pdf]

An Application of a Physiologically Based Pharmacokinetic Approach to Predict Ceftazidime Pharmacokinetics in Pregnant Population

Khaled Abduljalil[1*], Iain Gardner[1], Masoud Jamei [1]

[1]Certara Predictive Technologies Division, Level 2-Acero, 1 Concourse Way, Sheffield, S1 2BJ, United Kingdom

Supplementary document

Contents

Table S1. Ceftazidime PBPK model input parameters

Table S2. Collected clinical data on fraction of the dose excreted in the urine.

Figure S1. Fraction of ceftazidime excreted as unchanged ceftazidime in urine.

Figure S2. Fold change in systemic and renal clearance of ceftazidime at 10, 20, 30, and 40 GWs.

Table S1 Ceftazidime PBPK model input parameters

	**Parameter**	**value**	**Reference**
Physicochemical properties and binding	Mol Weight (g/mol)	546.580	Zhou et al., 2019 (1)
	log P	-3.750	
	Compound Type	Diprotic Acid	
	pKa 1	2.430	
	pKa 2	2.890	
	B/P	0.550	Default
	fup (Binding Protein)	0.9 (Human Serum Albumin)	Predicted and used as input
Absorption	Absorption (For intramuscular dosing only)		
	fa : Mean (%CV)	1 (0%CV)	assumed
	Ka (1/h): Mean (%CV)	2 (30%CV)	Adjusted to recover (2, 3)
	Tlag (h): Mean(%CV)	1.5 (30%CV)	
Distribution	Distribution Model	Full PBPK Model	
	Vss (L/kg)	0.20 (Predicted using Method 2 after (4))	
	Maternal Kp Scalar	1.0	
	Fetal Kp Scalar	1.0	
Elimination	Elimination option	Enzyme Kinetics	
	CL_R (L/h)	6.000	Zhou et al., 2019 (1)
	Biliary CLint (µL/min/million hepatocyte)	0.085 (30% CV)	Adjusted to recover Harding et al., 1983 (2)
**Pregnancy Model**			
Feo- placental model	CL_{PDM} (L/h/mL of placenta volume)	0.00137	Predicted (see text)
	CL_{PDF} (L/h/mL of placenta volume)	0.00137	Predicted (see text)
	fetal $CL_{Swallowing}$ (L/h/kg fetal weight): Mean (%CV)	0.00476 (30%)	see text
	fetal CL_R (L/h/kg fetal weight)	Predicted	GW-dependent (see text)
	$CL_{fetus <--> amniotic\ fluid}$ (L/h/kg fetal weight)	Predicted	GW-dependent (see text)

Mol Weight= molecular weight; logP=neutral species octanol: buffer partition coefficient; pKa= dissociation constant at logarithmic scale; BP=blood-to-plasma partition ratio; fup=fraction unbound in plasma; CL_R= renal clearance; CL_{PDM}=Passive diffusion clearance for Maternal-placental barrier; CL_{PDF}=Passive diffusion clearance for Placental-Fetal barrier

Table S2. Collected clinical data on fraction of the dose excreted in the urine from healthy adult non-pregnant subjects (Predicted mean (SD) 87(4) %) .

**Study design**			**Observed**		
Ref	**Dose**	**Sample Size (age range (years)**	**mean**	**SD**	**Ratio**
Harding et al 1983 (5)	0.5 g i.v.	4 M: 20-49 years	100		0.86
	1 g i.v.	4 M: 20-49 years	91.6		0.95
	1g i.m.	8 M: 20-49 years	82		1.06
Tjandramaga et al 1982 (6)	1g i.v.bolus (3min)	8 (0F): 22-24 years	96.2	5.5	0.90
	1g i.m.	8 (0F): 22-24 years	92.6	6.3	0.94
Harding et al 1983 (2)	0.5g i.v.	8 M: 20-49 years	85.6	8.2	1.01
	1 g i.v.	8 M: 20-49 years	85.1	4.8	1.02
	1 g 20 min i.v. inf	8 M: 20-49 years	83.7	7.65	1.04
	2 g 20-min i.v. inf	8 M: 20-49 years	87.1	4.2	1.00
	0.5g im	8 M: 20-49 years	85	6.5	1.02
	1g i.m.	8 M: 20-49 years	78.9	12	1.10
Kemmerich et al 1983 (7)	2g i.v. inf 20min	8 (50% F): 20-30yrs	72	12	1.22
Sommers et al 1983 (8)	1g i.v. bolus	16 (8F): 20-45 years	76	3	0.87
	1g i.m.	16 (8F): 20-45 years	74.7	3	0.86
Drusano et al., 1984 (9)	2g inf over 30min	6 (0F): 18 -35 years	80	10	1.10
Koyama et al. 1983 (3)	0.5g i.v. bolus	3 M: 31 years	91.2		1.04
	1g i.v. bolus	3 M: 31 years	89.3		0.97
	1g i.v. inf for 1 h	3 M: 27-29 years	89.6		0.96
	1g i.v. bolus	3 M: 23-27 years	95		1.09
	2g i.v. inf for 1 h	3 M: 27-29 years	90.2		0.96
	2g i.v. inf for 2 h	3 M : 23-38 years	93.3		0.93
	0.5g im	3 M: 23-35 years	91.3		0.94
Saito et al., 1994(10)	1g i.v. inf for 1 h	6 M: 20-23 years	91	5.1	0.95

i.v.=intravenous; i.m. =intramuscular; inf=infusion ; M= male; F=female.

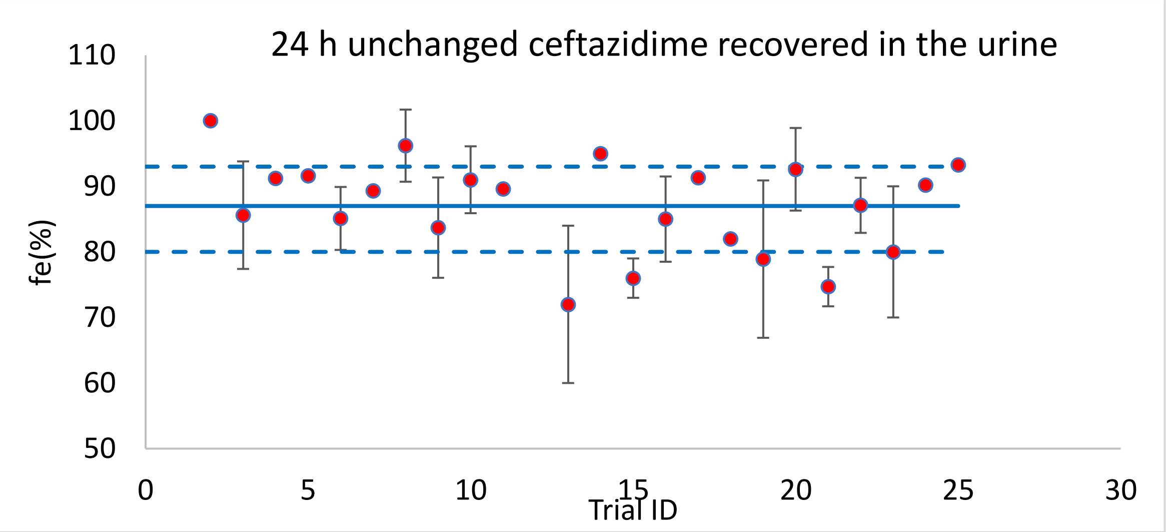

Figure S1. Fraction of ceftazidime excreted as unchanged ceftazidime in urine. Horizontal lines represent the PBPK model results (solid= mean; dashed= 5th and 95th percentiles) circles represent reported mean values in different clinical trials with error bars represent standard deviation.

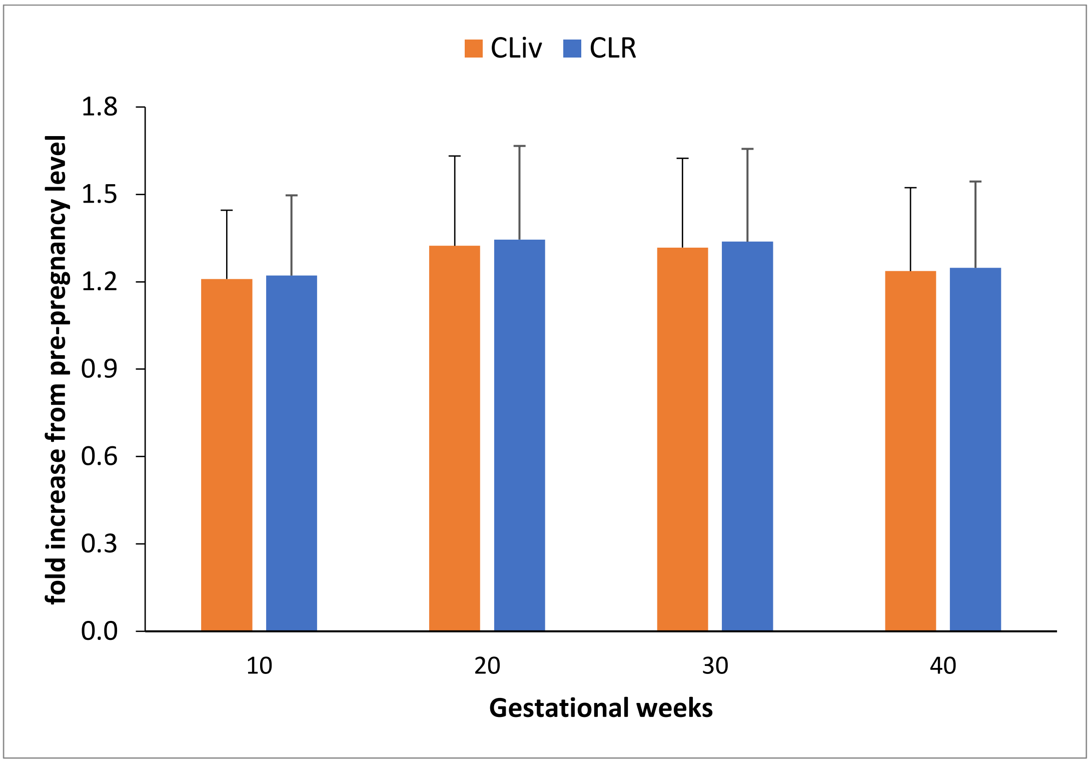

Figure S2. Fold change in systemic and renal clearance of ceftazidime at 10, 20, 30, and 40 GWs. Error bars represent standard error.

References

(1) Zhou, L., Tong, X., Sharma, P., Xu, H., Al-Huniti, N. & Zhou, D. Physiologically based pharmacokinetic modelling to predict exposure differences in healthy volunteers and subjects with renal impairment: Ceftazidime case study. *Basic Clin Pharmacol Toxicol* **125**, 100-7 (2019).

(2) Harding, S.M. & Harper, P.B. The pharmacokinetic behaviour of ceftazidime in man and the relationship between serum levels and the in vitro susceptibility of clinical isolates. *Infection* **11 Suppl 1**, S49-53 (1983).

(3) Koyama, M., Nakagawa, K., Takeda, K., Higo, K. & Okumura, K. Phase-one Clinical Study on Ceftazidime. *Chemotherapy* **31**, 146-55 (1983).

(4) Rodgers, T. & Rowland, M. Physiologically based pharmacokinetic modelling 2: predicting the tissue distribution of acids, very weak bases, neutrals and zwitterions. *J Pharm Sci* **95**, 1238-57 (2006).

(5) Harding, S.M., Monro, A.J., Thornton, J.E., Ayrton, J. & Hogg, M.I. The comparative pharmacokinetics of ceftazidime and cefotaxime in healthy volunteers. *J Antimicrob Chemother* **8 Suppl B**, 263-72 (1981).

(6) Tjandramaga, T.B., Van Hecken, A., Mullie, A., Verbesselt, R., De Schepper, P.J. & Verbist, L. Comparative pharmacokinetics of ceftazidime and moxalactam. *Antimicrob Agents Chemother* **22**, 237-41 (1982).

(7) Kemmerich, B., Warns, H., Lode, H., Borner, K., Koeppe, P. & Knothe, H. Multiple-dose pharmacokinetics of ceftazidime and its influence on fecal flora. *Antimicrob Agents Chemother* **24**, 333-8 (1983).
(8) Sommers, D.K., Walters, L., Van Wyk, M., Harding, S.M., Paton, A.M. & Ayrton, J. Pharmacokinetics of ceftazidime in male and female volunteers. *Antimicrob Agents Chemother* **23**, 892-6 (1983).
(9) Drusano, G.L. *et al.* Comparison of the pharmacokinetics of ceftazidime and moxalactam and their microbiological correlates in volunteers. *Antimicrob Agents Chemother* **26**, 388-93 (1984).
(10) Saito, A., Nakamichi, N., Obara, M., Watanabe, Y., Sakamoto, H. & Terakawa, M. Pharmacokinetics and serum bactericidal titers of FK037 and ceftazidime in healthy volunteers. *Chemotherapy* **42**, 114-28 (1994).
